# Training immunophenotyping deep learning models with the same-section ground truth cell label derivation method improves virtual staining accuracy

**DOI:** 10.3389/fimmu.2024.1404640

**Published:** 2024-06-28

**Authors:** Abu Bakr Azam, Felicia Wee, Juha P. Väyrynen, Willa Wen-You Yim, Yue Zhen Xue, Bok Leong Chua, Jeffrey Chun Tatt Lim, Aditya Chidambaram Somasundaram, Daniel Shao Weng Tan, Angela Takano, Chun Yuen Chow, Li Yan Khor, Tony Kiat Hon Lim, Joe Yeong, Mai Chan Lau, Yiyu Cai

**Affiliations:** ^1^ School of Mechanical and Aerospace Engineering, College of Engineering, Nanyang Technological University, Singapore, Singapore; ^2^ Institute of Molecular and Cell Biology, Agency for Science, Technology and Research, Singapore, Singapore; ^3^ Translational Medicine Research Unit, Medical Research Center Oulu, Oulu University Hospital, and University of Oulu, Oulu, Finland; ^4^ School of Electrical and Electronics Engineering, Nanyang Technological University, Singapore, Singapore; ^5^ Division of Medical Oncology, National Cancer Centre, Singapore, Singapore; ^6^ Department of Anatomical Pathology, Division of Pathology, Singapore General Hospital, Singapore, Singapore; ^7^ Bioinformatics Institute, Agency for Science, Technology and Research, Matrix, Singapore, Singapore; ^8^ Singapore Immunology Network, Agency for Science, Technology and Research, Immunos, Singapore, Singapore

**Keywords:** virtual staining, CD3, Pix2Pix generative adversarial network (P2P-GAN), tumor-infiltrating lymphocytes (TILs), hematoxylin and eosin (H&E), ground truth cell label, deep learning

## Abstract

**Introduction:**

Deep learning (DL) models predicting biomarker expression in images of hematoxylin and eosin (H&E)-stained tissues can improve access to multi-marker immunophenotyping, crucial for therapeutic monitoring, biomarker discovery, and personalized treatment development. Conventionally, these models are trained on ground truth cell labels derived from IHC-stained tissue sections adjacent to H&E-stained ones, which might be less accurate than labels from the same section. Although many such DL models have been developed, the impact of ground truth cell label derivation methods on their performance has not been studied.

**Methodology:**

In this study, we assess the impact of cell label derivation on H&E model performance, with CD3^+^ T-cells in lung cancer tissues as a proof-of-concept. We compare two Pix2Pix generative adversarial network (P2P-GAN)-based virtual staining models: one trained with cell labels obtained from the same tissue section as the H&E-stained section (the ‘same-section’ model) and one trained on cell labels from an adjacent tissue section (the ‘serial-section’ model).

**Results:**

We show that the same-section model exhibited significantly improved prediction performance compared to the ‘serial-section’ model. Furthermore, the same-section model outperformed the serial-section model in stratifying lung cancer patients within a public lung cancer cohort based on survival outcomes, demonstrating its potential clinical utility.

**Discussion:**

Collectively, our findings suggest that employing ground truth cell labels obtained through the same-section approach boosts immunophenotyping DL solutions.

## Introduction

Tissue-based multi-marker assays, such as multiplex immunohistochemistry (mIHC), are useful immunophenotyping tools by identifying molecular signatures. These signatures are crucial for cancer classification (1) and characterizing immune cells in terms of lineage and functional states (2, 3). However, the widespread adoption of these techniques is impeded by multiple factors, including limited access to specialized equipment, the need for skilled operators and extended turnaround times (4, 5). Thus, hematoxylin and eosin (H&E)-based deep learning (DL) prediction models have been developed as a viable alternative approach (6, 7). Given that H&E staining is low-cost and routinely performed in histology laboratories, integrating H&E-based prediction models into existing diagnostic workflows can be achieved with relative ease. This approach has the potential to revolutionize the field of immunotherapy by enabling accurate prediction of potential treatment response, especially since not all immunotherapy-treated patients respond well to the treatment (8, 9).H&E-based prediction models typically derive ground truth cell labels from chromogenic IHC-stained sections adjacent to a H&E-stained sections (‘serial-section’), assuming that cell locations are preserved across both sections (10–13). Yet, the gap between two serial sections likely hampers precise cell-to-cell mapping; moreover, manual sample preparation and heat fixation in conventional IHC can distort the sample and introduce artifacts (14). On the contrary, same-section mIHC (i.e., H&E and biomarker staining on the same tissue section) measures multiple markers to generate same-section ground truth labels. Additionally, mIHC enables a comprehensive analysis of the tumor microenvironment (TME) by quantifying multiple cell markers within the same tissue section (4); detailed TME characterization is crucial to predict if patients would respond to immunotherapy. For instance, the simultaneous quantification of the immune markers CD3, CD4, CD8, cytokeratin, PD-1, and CTLA-4 within the same tissue space reveals the intricacies of tumor-immune interactions (15, 16).

In this study, we demonstrate that a Pix2Pix generative adversarial network (P2P-GAN) model yields higher prediction accuracy for CD3^+^ T-cells trained on labels from the same tissue section, as opposed to labels from serial sections. Furthermore, we also tested the significance of the predicted CD3^+^ T-cells in the prognosis of lung cancer patients (17–19), which revealed that the same-section P2P-GAN model gave a more reliable prognosis than the serial-section P2P-GAN model. These findings underscore the critical role of accurate cell labeling in the development of effective immunotyping DL models.

## Materials and methods

### Cohorts

This study was conducted using tissue samples obtained from two in-house cohorts and one public cohort of lung cancer patients (**Table 1**). The training cohort (one of our in-house cohorts) comprises of formalin-fixed, paraffin-embedded (FFPE) lung carcinoma tissues collected from 57 patients, arranged in a tissue microarray (TMA), with one TMA core per patient, which was prepared at the Department of Anatomical Pathology of Singapore General Hospital (Agency of Science, Technology and Research; IRB numbers: 2021–161, 2021–188, 2021–112). The tissue sections were stained with H&E, and immunolabeled with an anti-CD3 antibody and 4’,6-diamidino-2-phenylindole (DAPI), with the latter two markers detected using mIHC, at the Institute of Molecular and Cell Biology at the Agency for Science, Technology and Research, Singapore. Two training datasets, namely the same-section and the serial-section datasets, were generated from the above training cohort as follows. In the same-section dataset, 57 H&E and mIHC image pairs were generated from the same tissue sections of the 57 patients. In the serial-section dataset, a separate set of H&E images were generated using tissue sections adjacent to the tissue sections used for the mIHC staining. All the TMA cores from both datasets were used for training.

**Table 1:** Characteristics of cohorts used for study.

Dataset type	Number of patients	Cores per patient	Image modalities	Tissue format	Image size (pixel-by-pixel)
Fluorescent mIHC training dataset (same- and serial-section)	57	1	H&E and mIHC image pairs	TMA cores	3228x3228
IHC testing dataset (same-section)					
48	1	H&E and IHC image pairs	TMA cores	Approximately 4000 x 4000	
Onco-SG testing dataset (20)	204	1–3	H&E	Region of interest in resected tissues	1792x768

To evaluate model performance, a second in-house cohort and a public cohort were used (**Table 1**). The in-house testing cohort, termed the IHC cohort, comprised CD3-stained chromogenic IHC images along with H&E images (20× magnification) generated from the same section. This IHC cohort is a separate lung carcinoma cohort of patients from the training cohort. The public cohort dataset, termed the Onco-SG cohort, consisted of H&E-stained images (20× magnification) obtained from the Singapore Oncology Data Portal (OncoSG). The corresponding patient survival data were also downloaded.

### Tissue staining

For the IHC cohort, the FFPE tissues were sectioned (4 µm thickness) and heat-fixed at 65°C for 5 min before being manually stained with hematoxylin (Epredia, Fisher Scientific, Porto Salvo, Portugal) and eosin (Epredia, Fisher Scientific, Gothenburg, Sweden). The H&E image was then acquired using the Axioscan.Z7 Slide Scanner (Zeiss, Oberkochen, Germany). Using the same H&E slide, the tissue section underwent decolorization via xylene, decreasing concentrations of ethanol and water. Then, the decolorized slide was subjected to chromogenic IHC staining. The slide was treated with an anti-CD3 primary antibody (Dako #A0452, Santa Clara, CA, USA) using the Leica Bond Max autostainer (Leica Biosystems, Melbourne, Australia) and the Bond Refine Detection Kit (Leica Biosystems) as previously described (21). The post-H&E IHC slide was then scanned using the Axioscan.Z7 Slide Scanner (Zeiss).

For the training cohort, fluorescent mIHC staining was performed on the FFPE tissue sections (4 µm thickness) using the Bond Max autostainer (Leica Biosystems), the Bond Refine Detection Kit (Leica Biosystems), and the Opal 6-Plex Detection Kit for Whole Slide Imaging (Akoya Biosciences, Marlborough, MA, USA) as previously described (21). Briefly, FFPE tissue sections were subjected to repeated cycles of heat-induced epitope retrieval, anti-CD3 primary antibody (Dako #A0452), anti-rabbit poly-HRP-IgG secondary antibody (Ready-to-use; Leica Biosystems), and Opal tyramide signal amplification reagent (Akoya Biosciences). Spectral DAPI (Akoya Biosciences) was then applied as the nuclear counterstain. mIHC images were captured using the Vectra 3 Automated Quantitative Pathology Imaging System (Akoya Biosciences). After scanning, to generate the same-section dataset, the mIHC slides were subjected to H&E staining, and rescanned using Axioscan.Z1 Slide Scanner (Zeiss). To generate the serial-section dataset, serial sections of FFPE tissue were stained directly with H&E and scanned with the Axioscan.Z1 Slide Scanner (Zeiss).

### Ground truth cell labeling of H&E images with fluorescent mIHC images

The fluorescent mIHC image was processed as follows. First, the DAPI channel was subjected to nuclear segmentation by the fluorescence StarDist model (22), generating a nuclear mask. Next, the CD3 channel was also processed with the fluorescence-trained StarDist model (22) to extract strong CD3^+^ signals. To determine the optimum arbitrary pixel intensity value for the generation of a binary CD3 mask, visual evaluation of CD3 positive signals using a range of pixel intensity values (between 0–255) was conducted by histologists. The optimum threshold of 50 was selected and applied to obtain a binary CD3 positive signal mask. Then, the CD3 mask was applied to the nuclear mask with the logical ‘AND’ operator from the Python package Numpy. Nuclei present in the same space as the positive signals in the CD3 mask were labeled as CD3^+^.

To transfer the cell labels from the processed mIHC image to the H&E image, the H&E image was first converted into a nuclear mask via nuclear segmentation by the H&E-trained StarDist model (22). The CD3^+^ cells identified in the mIHC image were matched to the closest nuclei in the H&E image generated from the same (post-mIHC H&E staining) or a serial tissue section (designated the same-section and serial-section datasets, respectively). To account for the membranous CD3 signals, the StarDist-generated CD3^+^ T-cell mask was dilated using the dilate function of OpenCV (kernel size 5).

### Ground truth cell labeling of H&E images with IHC images

For IHC images, each image was first subjected to color deconvolution by the deconvolution function from the scikit-image Python package (23). Next, to determine CD3 signal localization, a threshold of 100 was applied to the 3,3′-diaminobenzidine (DAB) stain channel, where pixel intensity values at 100 and above are classified as positive CD3 signals. This threshold value, chosen from a range between 0–255 (typical pixel intensity values in an image) was verified by histologists through visual inspection of the IHC images. A binary mask, where 1 indicates CD3 detection and 0 indicates otherwise, was subsequently obtained. The CD3 mask was then overlaid onto the nuclei segmented in the paired H&E image from the serial section to identify CD3^+^ T-cells (ground truth cell labels) according to the same procedure as that used for the mIHC dataset.

### RGB image tile generation

The H&E image was deconvoluted into hematoxylin and eosin channels with the color deconvolution and normalization functions from the scikit-image Python package. The minimum-maximum normalization function would also account for the intensity differences, especially those observed in post-mIHC H&E stained images. For the ground truth CD3^+^ cell labels from fluorescent mIHC images, Gaussian noise (kernel size 101, standard deviation ~11.875) was added using the Gaussian Blur function from the Python package OpenCV to increase the spread of the CD3^+^ signals while keeping the maximum intensity centered. This step is necessary for the GAN model to effectively learn the spatial distribution of CD3^+^ signals. The three images, i.e. the Hematoxylin and Eosin channels as well as the CD3^+^ signals were then stacked together into an RGB image using OpenCV’s merge function, with processed CD3^+^ signals in the R(ed) channel, hematoxylin signals in the B(lue) channel, and eosin signals in the G(reen) channel (**Supplementary Figure 1**). Images prepared for model inference contain data only in the G and B channels and the R channel will be populated with predictions from the P2P-GAN model.

### Obtaining predicted cell labels from GAN-generated images

To test our hypothesis, we chose to build P2P-GAN models that can predict CD3^+^ T-cells, considering the ease of visualization from the virtual staining capabilities of P2P-GAN (24–27). The predicted CD3 signals are extracted from the Red channel of the RGB image generated by the P2P-GAN model. The Gaussian noise is then removed from the predicted CD3 signals by applying a small (intensity > 2) binary threshold using OpenCV’s threshold function. This processed image is applied to the corresponding H&E image’s nuclear mask with the logical ‘AND’ operator from the Python package Numpy. Nuclei that have overlapping CD3 signals are determined to be CD3^+^ nuclei (**Supplementary Figure 2**). In cases of partial overlap, the nucleus is considered to be CD3^+^ if more than half of the nucleus overlaps with the CD3 signal, based on the pixel area. Comparing predicted nuclei coordinates with that of the ground truth nuclei coordinates helped calculate the overall model accuracy per tile for the training dataset and the held-out test subset or per TMA core for the IHC cohort.

### P2P-GAN model architecture

In a conventional GAN, there is a generative network that learns the feature representation of inputs like images and a discriminative network that evaluates them. The generative network is trained to ‘fool’ the discriminative network, thereby enabling unsupervised model learning. A conditional GAN, on the other hand, is an extension where the generation process is guided by additional conditions, such as specific input data (for instance, a H&E image in this context), which can lead to more controlled and targeted image generation. The P2P-GAN, a variation of a conditional GAN, is especially designed for image-to-image translation tasks. In this study, we adopted the P2P-GAN architecture reported by Isola et al. (28) in which a U-Net was used as the generator and a convolutional neural network (CNN) was used as the discriminator (**Supplementary Figure 3** and **Supplementary Tables 1**, **2**). During the learning process, the generator was presented with stain-deconvoluted H&E image patches, while the discriminator was presented with the ground truth image patches (i.e., stain-deconvoluted H&E images overlaid with mIHC-derived CD3^+^ T-cell information, RGB image patches). These images were then compared to images produced by the generator, resulting in a 30×30 matrix that was used to update both the generator and discriminator (**Supplementary Figure 3**).

### Model training

P2P-GAN models were trained using Tensorflow (ver. 2.4). Models trained on the same-section and serial-section training datasets are referred to as the same-section and serial-section models, respectively. Every image in the training dataset (**Table 1**) was resized to 3228×3228 pixels, using padding to maintain a consistent size across all images in a perfect square shape, facilitating subsequent image tiling. These resized images were then divided into 169 patches, each measuring 256×256 pixels, for a total of 9633 patches.

Patches were flagged if they did not contain significant CD3^+^ regions, which we defined arbitrarily as image patches where at least 10% patch area is occupied by CD3^+^ staining). For the main pair of same-section and serial-section models, flagged patches were excluded from the dataset. This was followed by a split of 90% randomly selected patches for model training (6912 patches for the same-section dataset and 4,050 for the serial-section dataset) and the remaining 10% patches that were kept for model testing (691 patches for the same-section dataset and 405 for the serial-section dataset), which is referred as the held-out subset.

A further two pairs of same-section and serial-section models were trained on matched dataset sizes. The first pair was trained on datasets that each contained 3646 patches, which is the minimum number of patches containing CD3^+^ regions between the two datasets. They were then tested on 405 patches. The second pair was trained on datasets that each contained 8670 patches, and tested on 961 patches, which when added together constitute the full size of the dataset.

During training, the generator and discriminator engage in an adversarial process to mitigate their respective losses. The overall objective is to find a balance between the two conflicting goals, for the generator to produce outputs nearly identical to the real (ground truth) images, and for the discriminator to accurately distinguish between the generator’s images and the real images. Overall, there are four different types of losses to be minimized: (1) LOSS 1, which measures the mean absolute difference between the generated image by the generator and the ground truth image, aiming to refine the generator network; (2) LOSS 2, also known as the Intersection over Union (IoU) loss, which evaluates the overlap between the predicted CD3^+^ regions and the actual ground truth CD3^+^ regions, ensuring positioning accuracy; (3) LOSS 3 and (4) LOSS 4, which measure the discrepancy between the 30×30 feature matrices output by the discriminator and the corresponding 30×30 target matrices: one consisting entirely of zeros, representing the ideal discriminator output when analyzing generated (fake) images, and the other filled with ones, representing the ideal discriminator output when analyzing real images. These differences are assessed using binary cross-entropy. This mechanism facilitates the evaluation of the discriminator’s ‘lack of capability’ and ‘capability’, respectively. Specifically, LOSS 3 provides dual feedback, assessing both the generator’s ability to create convincing images and the discriminator’s ability to differentiate between real and fake images. LOSS 4, on the other hand, offers feedback solely to the discriminator, pinpointing its accuracy in identifying generated images (**Supplementary Figure 3**). All models were trained using a batch size of 350, but differed in their optimization settings, specifically in the number of epochs and the regularization values for LOSS 2. The same-section model was initially optimized over 300 epochs with a LOSS 2 regularization value set at 100, and then further trained for an additional 150 epochs with an increased regularization value of 250. In contrast, the serial-section model started with a LOSS 2 regularization value of 250 for the initial 300 epochs, before the regularization value was increased to 500 for the subsequent 150 epochs. For all models, the regularization value for LOSS 1 was consistently maintained at 100. Due to the use of the hyperbolic tangent activation function in the generator, the input image patches were required to be normalized from the original range of 0 to 1 to a new range of -0.5 to 0.5. For analysis purposes, the output images generated during inference, were subsequently rescaled back to the original range of 0 to 1 range.

### Patient stratification for Onco-SG cohort

Alongside model prediction, two pathologists (Y.Z.X. and J.P.V.) conducted guided visual quantification of tumor-infiltrating lymphocytes (TILs). Using Qupath (29), an open-source software for digital pathology, they reviewed all the H&E images obtained from the Onco-SG repository and visually evaluated the percentage of TILs in each sample. The patients were then stratified into two groups: above average or below average model-predicted CD3^+^ T-cell counts or pathologist-defined TIL counts. For patients with multiple images, the mean percentages of CD3^+^ T-cells or TILs were first calculated.

### Statistical analysis

To compare the predicted CD3+ T-cell or CD3- cell counts with the corresponding ground truth cell counts, Pearson’s correlation analysis was performed using the *pearsonr* function from the Python package SciPy. The best-fit line was drawn with the Huber’s robust regression model using the HuberRegressor function from the Python package scikit-learn. To evaluate the relationship between model-predicted CD3^+^ T-cell counts and the pathologist-assessed TIL scores, which are ordinal in nature, Spearman’s correlation test was conducted, employing the *spearmanr* function from the Python package SciPy. Survival analysis was performed for patient groups stratified based on the percentage of model-predicted CD3^+^ T-cells relative to total cells (subsequently referred as CD3^+^ T-cell density), and or pathologist-determined TIL scores, using R programming. Keplan-Meier curves were generated for both the above-average and below-average predicted %CD3^+^/TIL groups, employing the *survival* and *survminer* R packages. The differentiation between these groups was assessed using the log-rank test to evaluate binary survival outcomes within a five-year period. The Cox-Proportional Hazards regression model was used to calculate the hazard ratio between the stratified groups. A significant difference between the groups and a low hazard ratio would suggest a strong association between the predicted %CD3^+^ and patient survival outcomes.

## Results

### Model performance evaluation using training samples

**Figure 1:**
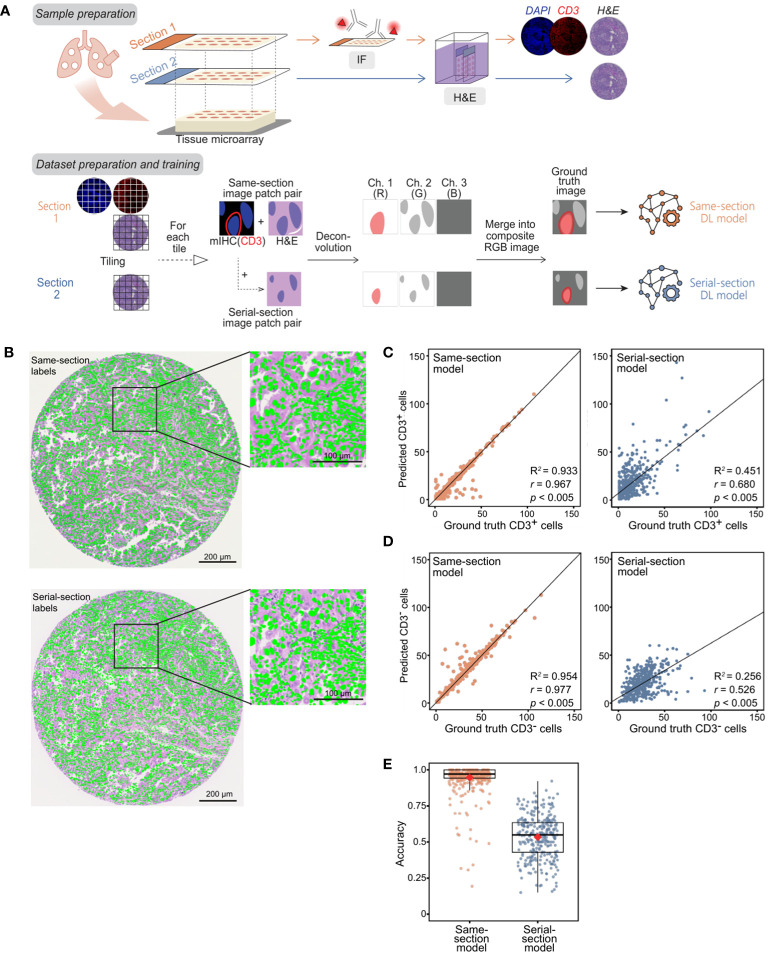
Development of P2P-GAN models trained on same-section and serial-section CD3^+^ fluorescent mIHC staining. **(A)** Preparation of samples and ground truth for both serial-section and same-section datasets followed by the construction and training of two P2P-GAN DL models utilizing the serial-section and same-section datasets. **(B)** An example of manually aligned mIHC DAPI channel (green) on the corresponding same-section and serial-section H&E image. **(C-E)** Model performance evaluation using the randomly selected held-out samples from the corresponding training cohorts (i.e., same-section and serial-section datasets, respectively). **(C)** CD3^+^ cell counts predicted by same-section and serial-section models compared with ground truth cell counts acquired from respective mIHC using Pearson’s correlation analysis (*r* and *p* values shown). Best-fit lines and R^2^ values obtained with Huber’s regression model are shown. **(D)** CD3^-^ cell counts predicted by same-section and serial-section models compared with ground truth cell counts acquired from respective mIHC using Pearson’s correlation analysis (*r* and *p* values shown). Best-fit lines and R^2^ values obtained with Huber’s regression model are shown. **(E)** Accuracy of same-section and serial-section model predictions. The boxplot shows the interquartile range (box), with the maximum values within 1.5 interquartile range from the upper and lower quartiles marked by the upper and lower whiskers, respectively. The red diamonds mark the mean values.

We trained the model on 256×256 pixel (56.32×56.32 μm) RGB cell image patches obtained from H&E-stained images of lung cancer samples, with the RGB layers being hematoxylin, eosin, and CD3^+^ positivity mask (**Figure 1A**). The CD3^+^ positivity masks were obtained from either same-section fluorescent mIHC or serial-section mIHC image (**Figure 1A, B**). As a sanity check, we assessed the models’ performance on their respective training datasets; specifically, the same-section model on the same-section training dataset, and similarly for the serial-section model (6912 patches for the same-section and 4000 for the serial-section datasets produced after filtering for CD3^+^ cell abundance). The cell counts predicted by both the same-section and serial-section models for CD3^+^ and CD3^-^ cells were closely aligned with the counts quantified by mIHC (considered as ground truth), exhibiting a high Pearson’s correlation coefficient (*r*) greater than 0.95, with a significance level *p* < 0.005 and an R^2^ score of greater than 0.95 with the best-fit line from the regression model (**Supplementary Figure 4A, B**), showing that the models were adequately trained. The average accuracy for both models was notably high: 98.2% for the same-section model and 96.3% for the serial-section model (**Supplementary Figure 4C**).

### Performance comparison of same-section and serial-section models on the held-out training cohort

The models were evaluated on their respective held-out datasets, consisting of 691 patches from the same-section dataset and 405 from the serial-section dataset. While both models generated CD3^+^ and CD3^-^ cell counts that were comparable to ground truth counts quantified by mIHC when tested on their training images (*p* < 0.005, **Supplementary Figure 1A, B**), the same-section model outperformed the serial-section on the held-out dataset (**Figure 1C, D**). Specifically, the predicted counts from the same-section model corresponded more closely with the ground truth than those from the serial-section model, both for CD3^+^ cells (*r* = 0.967 vs. 0.648; *p* < 0.005 for both; R^2^ score = 0.933 vs 0.451; **Figure 1C**) and CD3^-^ cells (*r* = 0.977 vs. 0.526, *p* < 0.005 for both; R^2^ score = 0.954 vs 0.256; **Figure 1D**). The mean accuracy of the same-section model is closer to 1 compared to that of the serial-section model with respective values of 94.6% and 53.4% (**Figure 1E**).

### Performance comparison of same-section and serial-section models on an independent IHC cohort

**Figure 2:**
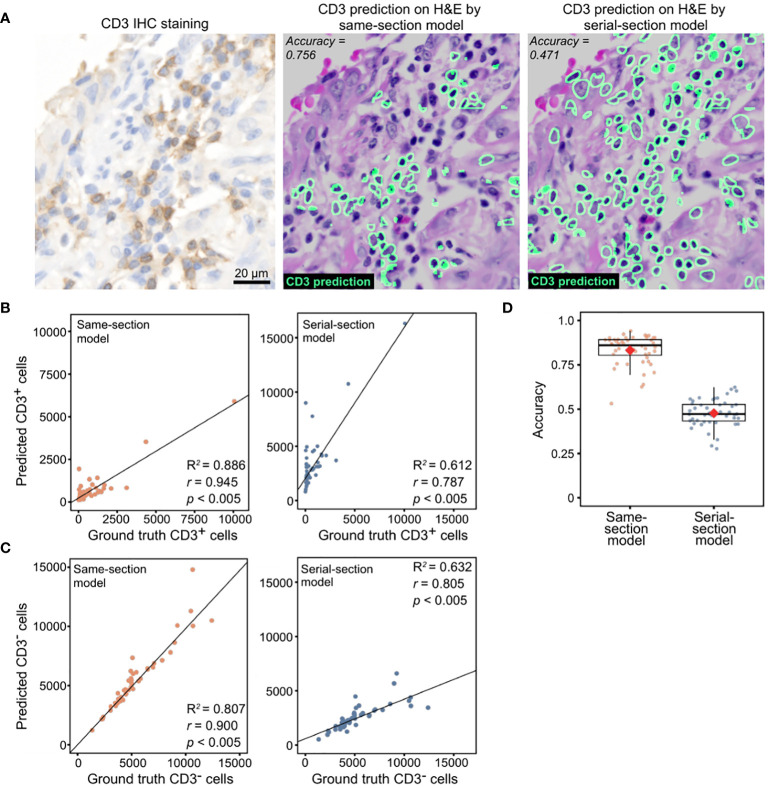
Model performance evaluation using an in-house lung cohort with IHC ground truth. **(A)** Representative images of CD3^+^ cell predictions by same-section and serial-section models with the corresponding CD3 IHC stain. **(B)** CD3^+^ cell counts predicted by same-section and serial-section models compared with ground truth cell counts acquired from CD3 IHC stain using Pearson’s correlation analysis. Diagonal dashed lines denote the best-fit line using Huber’s regression model. **(C)** CD3^-^ cell counts predicted by same-section and serial-section models compared with ground truth cell counts acquired from CD3 IHC stain using Pearson’s correlation analysis (*r* and *p* values shown). Best-fit lines and R^2^ values obtained with Huber’s regression model are shown. **(D)** Accuracy of same-section and serial-section model predictions. The boxplot shows the interquartile range (box), with the maximum values within 1.5 interquartile range from the upper and lower quartiles marked by the upper and lower whiskers, respectively. The red diamonds mark the mean values.

We subsequently evaluated the models using an independent lung cancer cohort (*n* = 48). The tissue sections in this cohort were stained for CD3 with chromogenic IHC, decolorized, then stained with H&E, generating the same-section ground truth. In agreement with the results obtained with the held-out dataset, the same-section model generated predictions that corresponded more closely with the IHC-derived ground truth for CD3^+^ T-cell counts (*r* = 0.900 vs. 0.805, *p* < 0.005 for both; R^2^ score = 0.807 vs 0.632; **Figure 2A, B**) and CD3^-^ cell counts (*r* = 0.945 vs. 0.787, *p* < 0.005 for both; R^2^ score = 0.886 vs 0.612; **Figure 2C**). Similar trends were observed in terms of mean accuracies (83.1 vs. 47.7; **Figure 2D**).

The same-section and serial-section models used so far were trained on differing dataset sizes resulting from retaining tiles containing at least 10% CD3^+^ cells (see Methods). To assess the impact of unequal training dataset size on model performance, we trained two more pairs of models: one pair of same- and serial-section models on a matched dataset size of 3646 tiles containing at least 10% CD3^+^ and another pair on the entire dataset of 8670 tiles that includes tiles without CD3^+^ cells. In concordance with results of the previous models (**Figure 2**), the same-section model trained on the 3646-tile dataset exhibited higher correlation with the IHC-derived ground truth for CD3^+^ cell counts (*r* = 0.907 vs. 0.835, *p* < 0.005 for both; R^2^ score = 0.821 vs 0.686; **Supplementary Figure 5A**) and CD3^-^ cell counts (*r* = 0.961 vs. 0.93, *p* < 0.005 for both; R^2^ score = 0.92 vs 0.861; **Supplementary Figure 5B**). When trained on the entire 8670-tile dataset that included tiles without CD3^+^ cells, the same-section model still outperformed the serial-section model (*r* = 0.884 vs. 0.805, *p* < 0.005 for both; R^2^ score = 0.779 vs 0.632; **Supplementary Figure 5C**) and CD3^-^ cell counts (*r* = 0.917 vs. 0.787, *p* < 0.005 for both; R^2^ score = 0.791 vs 0.612; **Supplementary Figure 5D**).

### Evaluation of prognosis based on model-predicted CD3^+^/CD3^-^ T-cell density

**Figure 3:**
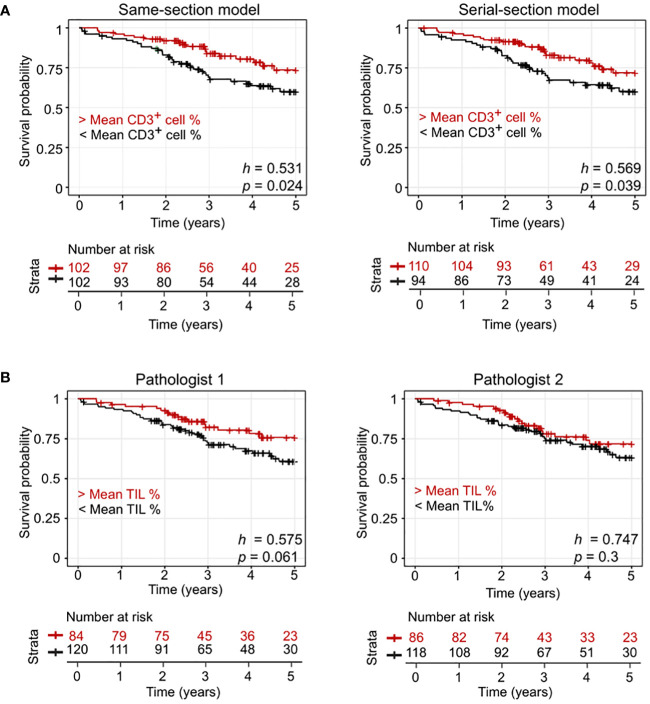
Model performance evaluation using an external lung cohort (Onco-SG). **(A)** Kaplen-Meier curves of patients with below average CD3^+^ counts and above average CD3^+^ counts predicted by same-section and serial-section models. **(B)** Kaplen-Meier curves of patients with pathologist-quantified below average tumor-infiltrating lymphocyte (TIL) counts and above average TIL counts. *h* values from the Cox-Proportional Hazard regression model and *p* values from log-rank test are shown.

We performed further evaluation of the models using data from an additional external dataset: the public Onco-SG cohort (*n* = 204) (**Table 1**). Our data indicated that the densities of CD3^+^ cells predicted by the models generally corresponded with pathologists’ scoring, with a positive Spearman’s rho (ρ) of at least 0.15 (*p* < 0.05) for both models, according to evaluations by two different pathologists (**Table 2**). The interobserver agreement between the two pathologists in TIL evaluation was low, with a kappa value of 0.178 (p < 0.005), indicating significant interobserver variability. Additionally, our analysis revealed that patient groups stratified by the model-predicted CD3^+^ cell densities demonstrated a significant association with patient 5-year survival rates (**Figure 3A**) in contrast to those stratified by pathologists’ scoring (**Figure 3B**). The same-section model demonstrated slightly higher significance in this respect (**Figure 3A**; same-section *p* = 0.024 vs. serial-section *p* = 0.039). The use of the average values of CD3^+^ cell densities and TIL % for stratification yielded unequal numbers of patients in each group.

**Table 2:** Spearman correlation of the prediction of CD3+ densities using the same and serial -section models with the manual TIL density scoring by two independent pathologists.

	Pathologist 1	Pathologist 2
Same-section model	P<0.05, (rho= 0.18)	P<0.05, (rho= 0.15)
Same-section model	P<0.05, (rho= 0.18)	P<0.05, (rho= 0.16)

We also checked the effect of dataset sizes with this cohort. When comparing the same-section and serial-section models trained on a matched dataset size of 3646 tiles containing at least 10% CD3^+^ cells, the same-section model outperformed the serial-section model in stratifying patients (**Supplementary Figure 6A**; same-section *p* = 0.036 vs. serial-section *p* = 0.039). The same was observed when comparing the models trained on the 8670 dataset, which contained tiles without CD3^+^ cells (**Supplementary Figure 6B**; same-section *p* = 0.015 vs. serial-section *p* = 0.02). This analysis demonstrated that our model provides prognostic value beyond what is achievable with manual TIL scoring on archived H&E images.

## Discussion

In this study, we developed two P2P-GAN virtual staining models to assess the impact of different approaches for deriving ground truth cell labels**—**same-section vs. serial-section**—**on the accuracy of predicting protein marker levels from cost-effective, digitized H&E images, with a focus on CD3^+^ T-cells in lung cancer patient tissues as the study model. Our results demonstrate that the model trained using the same-section approach consistently outperformed the model trained on serial-sections. This superior performance can likely be attributed to more accurate single-cell mapping between the mIHC and H&E sections, leading to more precise ground truth cell labeling, which in turn enhances model training. Crucially, our work also showcased the enhanced and consistent prognostic utility of model-predicted CD3^+^ T-cell density compared to traditional manual TIL scoring method, as evidenced by a weaker interobserver agreement value. Interobserver variability between pathologists could be a result of different training backgrounds and reporting habits, especially in a multi-institutional setting (30). Conversely, our proposed virtual staining model provides a more reproducible solution by being unaffected by human subjectivity and potential biases, thereby enhancing patient stratification and aiding in treatment decision-making. The results above have shown that training the model on ground truth cell labels derived from the same tissue section leads to better model performance. Aside from enabling same section staining, the fluorescent mIHC technique allows for the same-section model to be potentially developed as a generalized approach for integrating predictions of different cell types (e.g., CD8^+^ T-cells, CD68^+^ macrophages) or more refined cell types (e.g., CD3^+^CD8^+^) from various models in the same H&E space. This reduces the need for multiple tissue sections for analysis, as well as decreases the time and costs associated with staining procedures. Consequently, analysis of multi-markers or cell types becomes feasible on retrospective archived H&E slides. Collectively, these advancements could significantly enhance our understanding of the TME, making the identification of novel spatial biomarkers or therapeutic targets more streamlined and cost-effective.

While more studies are recognizing the importance of using same-section tissue to generate ground truth cell labels (31, 32), our study provides a comprehensive quantification of the impact on prediction accuracy, which has not been previously performed. Additionally, our study conducts prediction on H&E images instead of autofluorescence images (31) or IHC images (32), a gold standard stain used ubiquitously in many clinical laboratories for pathological diagnosis. Therefore, our DL pipeline has the potential for future assimilation into existing clinical practices and workflows.

Unlike the conventional GAN model, we proposed the P2P-GAN model for its image-to-image translation capability, allowing virtual staining of CD3^+^ signals within the input H&E image. To enhance the positional accuracy of predicted CD3^+^ signals, we incorporated the use of an IoU loss function, that evaluates overlap between ground truth CD3^+^ regions and the predicted CD3^+^ regions, in one of the loss functions. Additionally, considering that CD3 localizes to the cell membrane, we applied Gaussian blurring to the CD3 signals before overlaying them onto the corresponding cells in the H&E image. Collectively, these features ensure positional accuracy, when combined with the novel approach of ground truth cell labeling on the same section stained for H&E image, facilitate effective analysis of cellular interactions and comprehensive TME analysis based on cell types predicted from the H&E images.

While our proposed approach has yielded encouraging results, it is important to acknowledge its inherent limitations. Firstly, our current model is specifically trained for CD3^+^ T-cell prediction from H&E images and might require modifications and re-training to generalize effectively to other cell types or biomarkers. Additionally, its performance may be compromised when applied to tumor types other than lung cancer. Secondly, the application of our model is largely limited to high-quality digital slides of consistent quality; its performance may therefore be affected by variations in tissue preparation, staining, and imaging procedures which may vary significantly across different laboratories. Nevertheless, the clinical significance of our model has been validated using a publicly available dataset, which included sample images collected by different laboratories. Lastly, despite the overall robustness of our model, we noted some outliers in the predictions, indicating potential areas for improvement. The existence of these outliers suggests there are complex and unaddressed variables within biological samples which require further investigation. Future research endeavors should aim to unravel the reasons behind these outliers, refine the model, and include larger, more diverse datasets for improved generalizability and outlier management.

In conclusion, our study emphasizes the importance of using accurate ground truth cell labels, enabled by same-section molecular staining through mIHC. This approach represents a significant advancement in H&E-based predictive research and holds potential for clinical implementation. Moreover, our proposed approach allows the development of multiple virtual staining models from the same H&E slide, leveraging mIHC-quantified multiple protein markers (e.g., PD-1, CD3), reinforcing its potential as a robust technique in histopathology-driven immunophenotyping. By enabling the overlay of multiple markers or immunophenotype predictions within the same H&E space through our proposed P2P-GAN virtual staining approach, our method unveils exciting new possibilities for biomarker discovery and the advancement of therapeutic strategies.

## Data availability statement

The raw data supporting the conclusions of this article will be made available by the authors upon reasonable request. The codes can be found on GitHub, https://github.com/abubakrazam/Pix2Pix_TIL_H-E.git.

## Ethics statement

The studies involving humans were approved by Agency of Science, Technology and Research Human Biomedical Research Office. (IRB numbers: 2021-161, 2021-188, 2021-112). The studies were conducted in accordance with the local legislation and institutional requirements. Written informed consent for participation in this study was provided by the participants’ legal guardians/next of kin.

## Author contributions

AA: Data Curation, Formal analysis, Investigation, Methodology, Software, Validation, Visualization, Writing – original draft, Writing – review & editing. FW: Data Curation, Investigation, Methodology, Resources, Validation, Writing – original draft, Writing – review & editing. JV: Validation, Writing – review & editing. WY: Visualization, Validation, Writing – original draft, Writing – review & editing. YX: Validation, Writing – review & editing. BC: Validation, Writing – review & editing. JL: Investigation, Resources, Writing – review & editing. AS: Investigation, Methodology, Software, Writing – review & editing. DT: Resources, Writing – review & editing. AT: Resources, Writing – review & editing. CC: Resources, Writing – review & editing. LK: Resources, Writing – review & editing. TL: Resources, Writing – review & editing. JY: Conceptualization, Funding Acquisition, Resources, Writing – review & editing. YC: Conceptualization, Resources, Writing – review & editing. ML: Conceptualization, Data Curation, Formal analysis, Resources, Supervision, Validation, Visualization, Writing – original draft, Writing – review & editing.
