# Supplementary material for: Training immunophenotyping deep learning models with the same-section ground truth cell label derivation method improves virtual staining accuracy
Source: Front Immunol. 2024 Jun 28;15:1404640. doi: 10.3389/fimmu.2024.1404640 (PMC11239356; doi:10.3389/fimmu.2024.1404640)
Supplement: Supplementary file 1 [file DataSheet_1.pdf]

Supplementary Figure 1

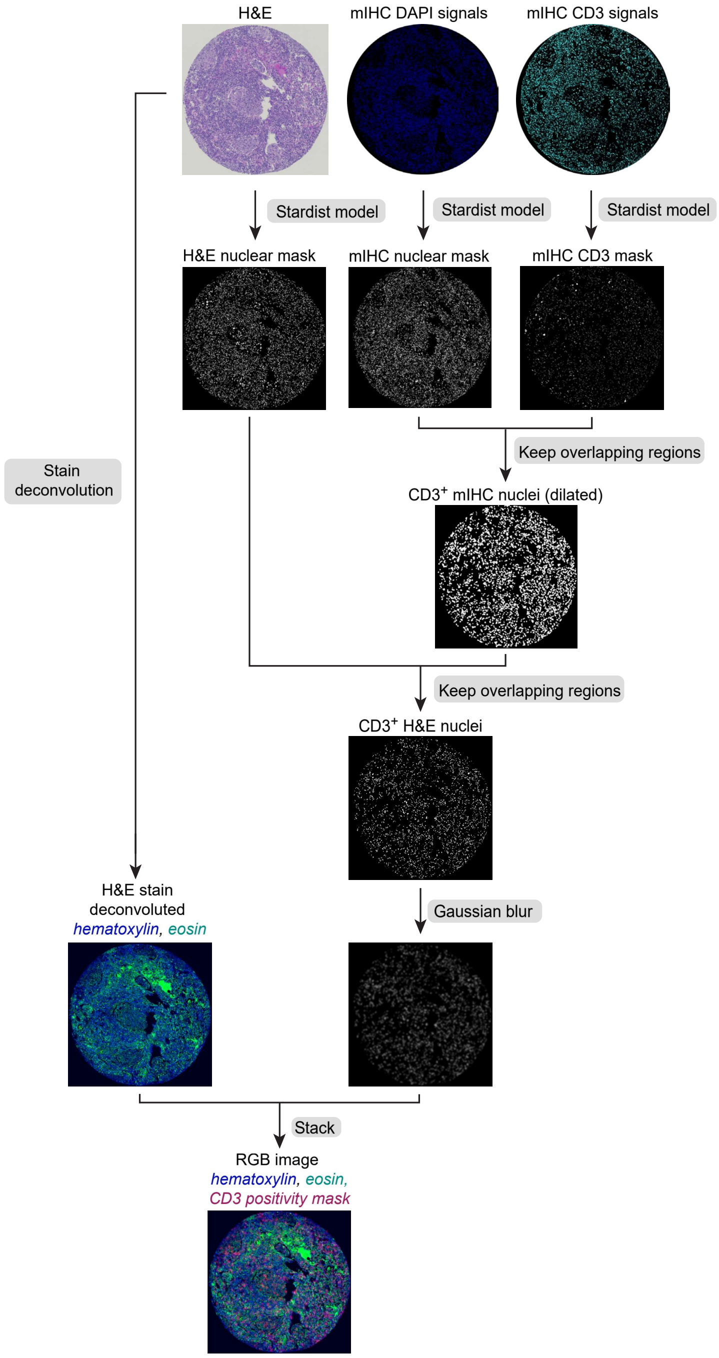

Supplementary Figure 1. Generation of training RGB images containing hematoxylin, eosin, and CD3 positivity mask. The H&E and mIHC images were first converted into masks via the StarDist model. The mIHC nuclear mask (obtained from the DAPI channel) was applied to the CD3 mask to identify $CD3^+$ nuclei. This was then applied to the H&E nuclear mask to identify $CD3^+$ nuclei present in the H&E image. Following Gaussian blurring, the $CD3^+$ H&E nuclear mask was stacked with separate hematoxylin and eosin channels that were obtained by stain deconvolution. The final stack is an RGB image, with $CD3^+$ mask in the R(ed channel), hematoxylin signals in the B(lue) channel, and eosin signals in the G(reen) channel. These RGB images are used for training and are the format of the P2P-GAN model output.

Supplementary Figure 2

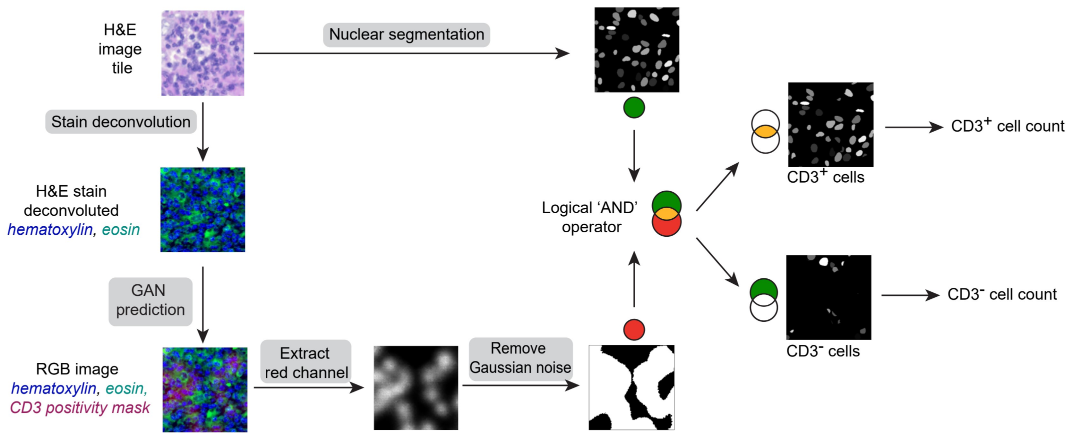

Supplementary Figure 2. Identification of $CD3^+$ and $CD3^-$ cells predicted by the proposed P2P-GAN models. This process involves extracting the model-predicted CD3 signals (in the red channel) and overlaying the detected signal onto nuclei detected in the H&E image with a logical 'AND' operator. Nuclei with overlapping CD3 signals are regarded as $CD3^+$ cells.

Supplementary Figure 3

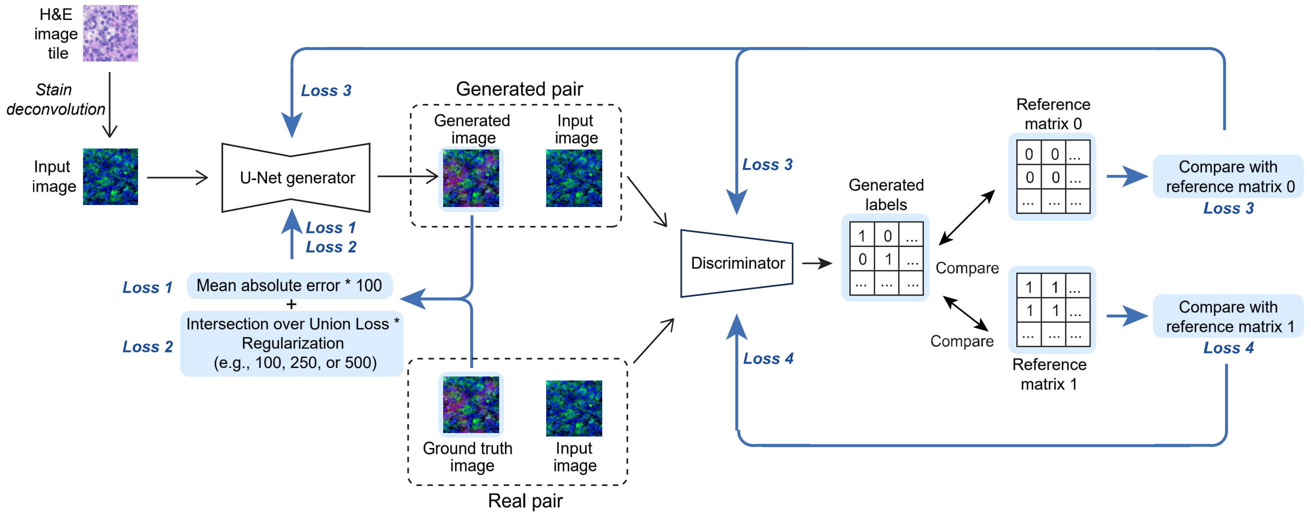

Supplementary Figure 3. P2P-GAN model architecture and parameter updating process during model training. The generator accepts the H&E image patch and generates (predicts) $CD3^+$ signals on the input image. The discriminator, which is the adversary to the generator, distinguishes the generator's output from the ground truth image, i.e., the RGB image patch with hematoxylin in the B(lue) channel, eosin in the G(reen) channel, and mIHC-derived $CD3^+$ signals in the R(ed) channel. The adversarial nature of the network drives the generator to produce better predictions, which are determined by the reference matrices, i.e., 30×30 matrices with 1s (representing ground truth images) and matrices with 0s (representing generated images). The binary-cross entropy loss maps the differences between the predictions and the reference matrices between the range of 0-1, with 0 denoting the highest similarity between either matrices. Poor predictions would bear close resemblance to the 0s matrix while good predictions should bear close resemblance to the ground truth, i.e., the 1s matrix.

Supplementary Figure 4

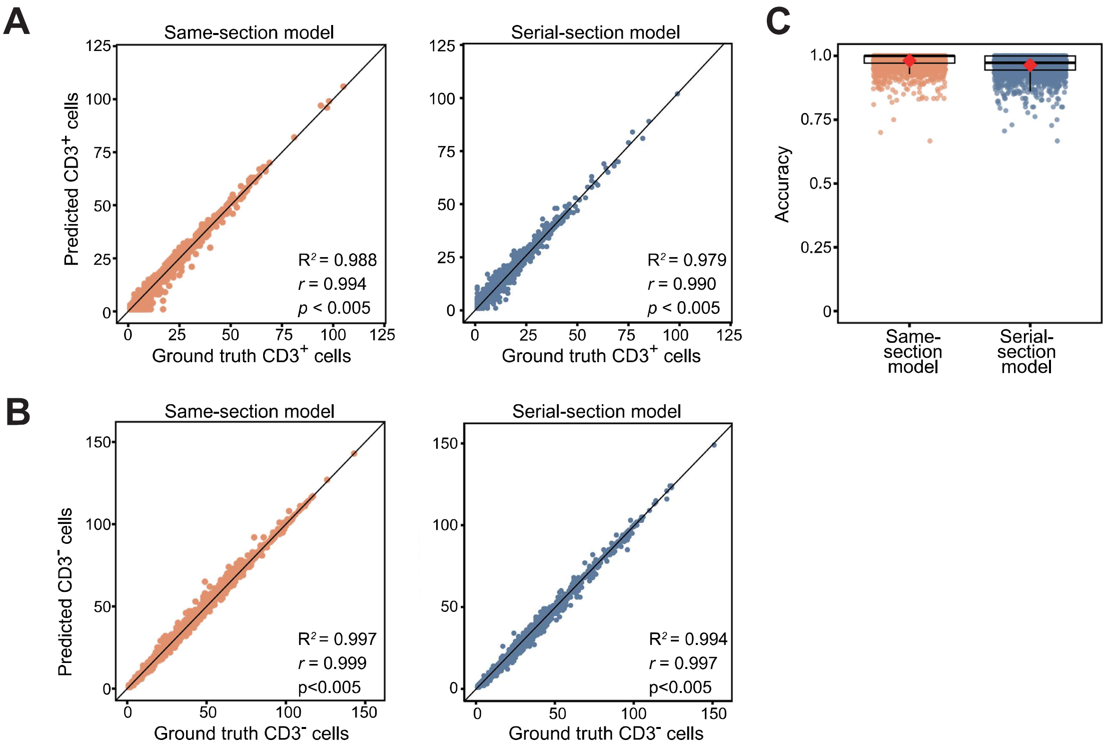

Supplementary Figure 4. Model performance evaluation using training data as a sanity check. **(A)** $CD3^+$ cell counts predicted by the same-section model on same-section training data (left) and the serial-section model on serial section training data (right). The predicted $CD3^+$ cell counts were compared with ground truth cell counts using Pearson's correlation analysis (*r* and *p* values shown). Best-fit lines and R^2 values obtained with Huber's regression model are shown. **(B)** Same as described in (A) but for $CD3^-$ cell counts. **(C)** Accuracy of same-section and serial-section model predictions. The boxplot shows the interquartile range (box), with the maximum values within 1.5 interquartile range from the upper and lower quartiles marked by the upper and lower whiskers, respectively. The red diamonds mark the mean values.

Supplementary Figure 5

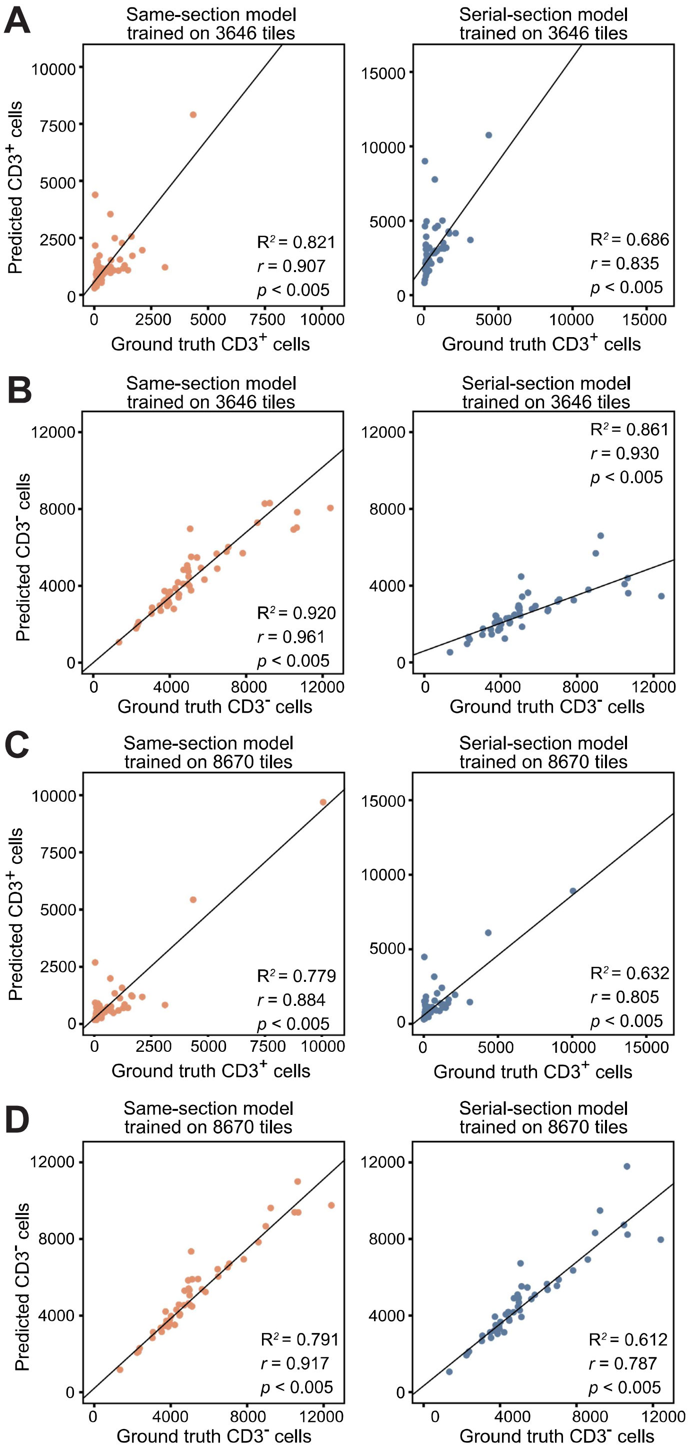

Supplementary Figure 5. Evaluation of training dataset size on fluorescent mIHC ground truth-trained model performance evaluation using an in-house lung cohort with chromogenic IHC ground truth. **(A)** $CD3^+$ cell counts predicted by same-section and serial-section models trained on mIHC datasets that each contain 3646 tiles (with $CD3^+$ cell abundance filtering) compared with ground truth cell counts acquired from CD3 IHC stain using Pearson's correlation analysis (*r* and *p* values shown). Best-fit lines and R^2 values obtained with Huber's regression model are shown. **(B)** Same as described in (A) but for $CD3^-$ cell counts. **(C)** $CD3^+$ cell counts predicted by same-section and serial-section models trained on fluorescence mIHC datasets that each contain 8670 tiles (without $CD3^+$ cell abundance filtering) compared with ground truth cell counts acquired from CD3 IHC stain using Pearson's correlation analysis (*r* and *p* values shown). Best-fit lines and R^2 values obtained with Huber's regression model are shown. **(D)** Same as described in (C) but for $CD3^-$ cell counts.

Supplementary Figure 6

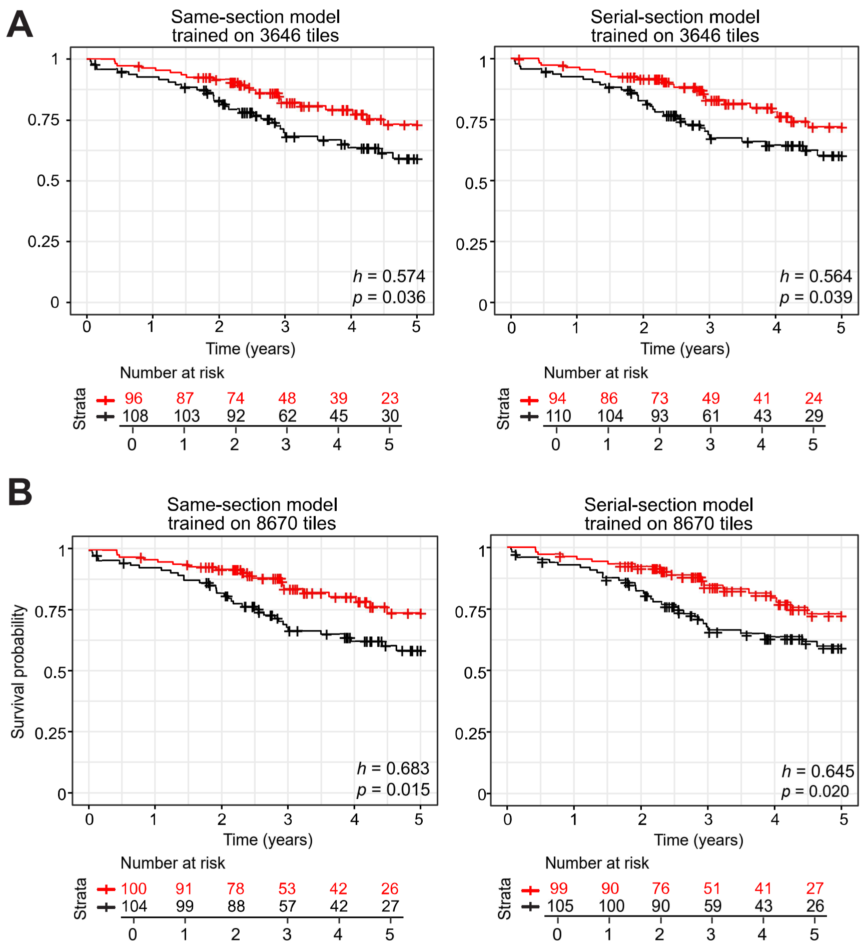

Supplementary Figure 6. Evaluation of training dataset size on fluorescent mIHC-trained model performance using an external lung cohort (Onco-SG). **(A)** Kaplen-Meier curves of patients with below average $CD3^+$ counts and above average $CD3^+$ counts predicted by same-section and serial-section models trained on mIHC datasets that each contain 3646 tiles (with $CD3^+$ cell abundance filtering). **(B)** Kaplen-Meier curves of patients with below average $CD3^+$ counts and above average $CD3^+$ counts predicted by same-section and serial-section models trained on fluorescence mIHC datasets that each contain 8670 tiles (without $CD3^+$ cell abundance filtering). *h* values from the Cox-Proportional Hazard regression model and p values from log-rank test are shown.
